# Supplementary material for: Baseline Characterization of the Gut Microbiota of Field and Colony Populations of Phlebotomus tobbi and Preliminary Assessment of the Anti-Leishmanial Activity of Cultivable Bacteria
Source: Pathogens. 2026 Jun 23;15(7):658. doi: 10.3390/pathogens15070658 (PMC13416292; doi:10.3390/pathogens15070658)
Supplement: Supplementary file 1 [file pathogens-15-00658-s001.zip › pathogens-4357168-File S1/Taxa2ASV_Decomposer/Family/f__Enterobacteriaceae/seqs.align.html]

```
seq62           AGTGGGGAATATTGCACAATGGGCGCAAGCCTGATGCAGCCATGCCGCGTGTATGAAGAA
seq59           AGTGGGGAATATTGCACAATGGGCGCAAGCCTGATGCAGCCATGCCGCGTGTATGAAGAA
seq24           AGTGGGGAATATTGCACAATGGGCGCAAGCCTGATGCAGCCATGCCGCGTGTgTGAAGAA
seq27           AGTGGGGAATATTGCACAATGGGCGCAAGCCTGATGCAGCCATGCCGCGTGTgTGAAGAA
seq61           AGTGGGGAATATTGCACAATGGGCGCAAGCCTGATGCAGCCATGCCGCGTGTgTGAAGAA
seq3            AGTGGGGAATATTGCACAATGGGCGCAAGCCTGATGCAGCCATGCCGCGTGTgTGAAGAA
seq14           AGTGGGGAATATTGCACAATGGGCGCAAGCCTGATGCAGCCATGCCGCGTGTgTGAAGAA
seq20           AGTGGGGAATATTGCACAATGGGCGCAAGCCTGATGCAGCCATGCCGCGTGTATGAAGAA
seq21           AGTGGGGAATATTGCACAATGGGCGCAAGCCTGATGCAGCCATGCCGCGTGTATGAAGAA
seq33           AGTGGGGAATATTGCACAATGGGCGCAAGCCTGATGCAGCCATGCCGCGTGTATGAAGAA
seq67           AGTGGGGAATATTGCACAATGGGCGCAAGCCTGATGCAGCCATGCCGCGTGTATGAAGAA
seq35           AGTGGGGAATATTGCACAATGGGCGCAAGCCTGATGCAGCCATGCCGCGTGTATGAAGAA
seq52           AGTGGGGAATATTGCACAATGGGCGCAAGCCTGATGCAGCCATGCCGCGTGTATGAAGAA
seq19           AGTGGGGAATATTGCACAATGGGCGCAAGCCTGATGCAGCCATGCCGCGTGTATGAAGAA
seq40           AGTGGGGAATATTGCACAATGGGCGCAAGCCTGATGCAGCCATGCCGCGTGTATGAAGAA
seq43           AGTGGGGAATATTGCACAATGGGCGCAAGCCTGATGCAGCCATGCCGCGTGTATGAAGAA
seq25           AGTGGGGAATATTGCACAATGGGCGCAAGCCTGATGCAGCCATGCCGCGTGTATGAAGAA
seq1            AGTGGGGAATATTGCACAATGGGCGCAAGCCTGATGCAGCCATGCCGCGTGTATGAAGAA
seq12           AGTGGGGAATATTGCACAATGGGCGCAAGCCTGATGCAGCCATGCCGCGTGTATGAAGAA
seq2            AGTGGGGAATATTGCACAATGGGCGCAAGCCTGATGCAGCCATGCCGCGTGTATGAAGAA
seq8            AGTGGGGAATATTGCACAATGGGCGCAAGCCTGATGCAGCCATGCCGCGTGTATGAAGAA
seq56           AGTGGGGAATATTGCACAATGGGCGCAAGCCTGATGCAGCCATGCCGCGTGTgTGAAGAA
seq32           AGTGGGGAATATTGCACAATGGGCGCAAGCCTGATGCAGCCATGCCGCGTGTATGAAGAA

seq62           GGCCTTCGGGTTGTAAAGcACTTTtgtCcGGaAaGAAatgGcTctGGTTAATAcCtggGg
seq59           GGCCTTCGGGTTGTAAAGTACTTTCAGtcGGGAGGAAGGtGtcagGGTTAATAACCctGa
seq24           GGCCTTCGGGTTGTAAAGcACTTTCAGCGaGGAGGAAGGgtAgtgtGTTAATAgCacatT
seq27           GGCCTTCGGGTTGTAAAGcACTTTCAGCGaGGAGGAAGGgttcagtGTTAATAgCactGT
seq61           GGCCTTCGGGTTGTAAAGcACTTTCAGCGaGGAGGAAGGtGgTGAGcTTAATAcgCTCaT
seq3            GGCCTTCGGGTTGTAAAGcACTTTCAGCGaGGAGGAAGGtGgTGAGcTTAATAcgCTCaT
seq14           GGCCTTCGGGTTGTAAAGcACTTTCAGCGaGGAGGAAGGtGgTGAacTTAATAcgtTCaT
seq20           GGCCTTCGGGTTGTAAAGTACTTTCAGCGGGGAGGAAGGCGATGtGGTTAATAACCgCGT
seq21           GGCCTTCGGGTTGTAAAGTACTTTCAGCGGGGAGGAAGGCGATGtGGTTAATAACCgCaT
seq33           GGCCTTCGGGTTGTAAAGTACTTTCAGCGGGGAGGAAGGCGATGcGGTTAATAACCgCGT
seq67           GGCCTTCGGGTTGTAAAGTACTTTCAGCGGGGAGGAAGGCGAcGgGGTTAATAACCctGT
seq35           GGCCTTCGGGTTGTAAAGTACTTTCAGCGGGGAGGAAGGCGAcGgGGTTAATAACCctGT
seq52           GGCCTTCGGGTTGTAAAGTACTTTCAGCGGGGAGGAAGGCGATGgGGTTAATAACCctGT
seq19           GGCCTTCGGGTTGTAAAGTACTTTCAGCGGGGAGGAAGGCGATaAGGTTAATAACCTtGT
seq40           GGCCTTCGGGTTGTAAAGTACTTTCAGCGGGGAGGAAGGCGgTGAGGTTAATAACCTCac
seq43           GGCCTTCGGGTTGTAAAGTACTTTCAGCGGGGAGGAAGGCGgTGAGGTTAATAACCTCGc
seq25           GGCCTTCGGGTTGTAAAGTACTTTCAGCGGGGAGGAAGGtGtTGAGGTTAATAACCTCag
seq1            GGCCTTCGGGTTGTAAAGTACTTTCAGtGGGGAGGAAGGCGATGgaGTTAATAgCtTCGT
seq12           GGCCTTCGGGTTGTAAAGTACTTTCAGtGGGGAGGAAGGCGATGAaGTTAATAgCtTCGT
seq2            GGCCTTCGGGTTGTAAAGTACTTTCAGtGGGGAGGAAGGCGATGAaGTTAATAgCtTCGT
seq8            GGCCTTCGGGTTGTAAAGTACTTTCAGtGGGGAGGAAGGCGAaGAGGTTAATAACCTttT
seq56           GGCCTTCGGGTTGTAAAGcACTTTCAGCGGGGAGGAAGGCGtTaAGGTTAATAACCTtGg
seq32           GGCCTTCGGGTTGTAAAGTACTTTCAGCGGGGAGGAAGGCGATaAGGTTAATAACCTtGT

seq62           tcgaTGACGgTACCgGaAGAAtAAGgACCGGCTAACTaCGTGCCAGCAGCCGCGGTAATA
seq59           CaATTGACGTTACCgaCAGAAGAAGCACCGGCTAACTCCGTGCCAGCAGCCGCGGTAATA
seq24           gcATTGACGTTACtCGCAGAAGAAGCACCGGCTAACTCCGTGCCAGCAGCCGCGGTAATA
seq27           tcATTGACGTTACtCGCAGAAGAAGCACCGGCTAACTCCGTGCCAGCAGCCGCGGTAATA
seq61           CaATTGACGTTACtCGCAGAAGAAGCACCGGCTAACTCCGTGCCAGCAGCCGCGGTAATA
seq3            CaATTGACGTTACtCGCAGAAGAAGCACCGGCTAACTCCGTGCCAGCAGCCGCGGTAATA
seq14           CaATTGACGTTACtCGCAGAAGAAGCACCGGCTAACTCCGTGCCAGCAGCCGCGGTAATA
seq20           CGATTGACGTTACCCGCAGAAGAAGCACCGGCTAACTCCGTGCCAGCAGCCGCGGTAATA
seq21           tGATTGACGTTACCCGCAGAAGAAGCACCGGCTAACTCCGTGCCAGCAGCCGCGGTAATA
seq33           CGATTGACGTTACCCGCAGAAGAAGCACCGGCTAACTCCGTGCCAGCAGCCGCGGTAATA
seq67           CGATTGACGTTACCCGCAGAAGAAGCACCGGCTAACTCCGTGCCAGCAGCCGCGGTAATA
seq35           CGATTGACGTTACCCGCAGAAGAAGCACCGGCTAACTCCGTGCCAGCAGCCGCGGTAATA
seq52           CGATTGACGTTACCCGCAGAAGAAGCACCGGCTAACTCCGTGCCAGCAGCCGCGGTAATA
seq19           CGATTGACGTTACCCGCAGAAGAAGCACCGGCTAACTCCGTGCCAGCAGCCGCGGTAATA
seq40           CGATTGACGTTACCCGCAGAAGAAGCACCGGCTAACTCCGTGCCAGCAGCCGCGGTAATA
seq43           CGATTGACGTTACCCGCAGAAGAAGCACCGGCTAACTCCGTGCCAGCAGCCGCGGTAATA
seq25           CaATTGACGTTACCCGCAGAAGAAGCACCGGCTAACTCCGTGCCAGCAGCCGCGGTAATA
seq1            CGATTGACGTTACCCGCAGAAGAAGCACCGGCTAACTCCGTGCCAGCAGCCGCGGTAATA
seq12           CGATTGACGTTACCCGCAGAAGAAGCACCGGCTAACTCCGTGCCAGCAGCCGCGGTAATA
seq2            CGATTGACGTTACCCGCAGAAGAAGCACCGGCTAACTCCGTGCCAGCAGCCGCGGTAATA
seq8            CGATTGACGTTACCCGCAGAAGAAGCACCGGCTAACTCCGTGCCAGCAGCCGCGGTAATA
seq56           CGATTGACGTTACCCGCAGAAGAAGCACCGGCTAACTCCGTGCCAGCAGCCGCGGTAATA
seq32           CGATTGACGTTACCCGCAGAAGAAGCACCGGCTAACTCCGTGCCAGCAGCCGCGGTAATA

seq62           CGGAGGGTGCAAGCGTTAATCGGAATTACTGGGCGTAAAGCGCACGCAGGCGGTCTGTcA
seq59           CGGAGGGTGCAAGCGTTAATCGGAATTACTGGGCGTAAAGCGCACGCAGGCGGTtgaTTg
seq24           CGGAGGGTGCAAGCGTTAATCGGAATTACTGGGCGTAAAGCGCACGCAGGCGGTtTGTTA
seq27           CGGAGGGTGCAAGCGTTAATCGGAATTACTGGGCGTAAAGCGCACGCAGGCGGTtTGTTA
seq61           CGGAGGGTGCAAGCGTTAATCGGAATTACTGGGCGTAAAGCGCACGCAGGCGGTCTGTcA
seq3            CGGAGGGTGCAAGCGTTAATCGGAATTACTGGGCGTAAAGCGCACGCAGGCGGTtTGTTA
seq14           CGGAGGGTGCAAGCGTTAATCGGAATTACTGGGCGTAAAGCGCACGCAGGCGGTtTGTTA
seq20           CGGAGGGTGCAAGCGTTAATCGGAATTACTGGGCGTAAAGCGCACGCAGGCGGTCTGTTA
seq21           CGGAGGGTGCAAGCGTTAATCGGAATTACTGGGCGTAAAGCGCACGCAGGCGGTCTGTTA
seq33           CGGAGGGTGCAAGCGTTAATCGGAATTACTGGGCGTAAAGCGCACGCAGGCGGTCTGTTA
seq67           CGGAGGGTGCAAGCGTTAATCGGAATTACTGGGCGTAAAGCGCACGCAGGCGGTCTGTTA
seq35           CGGAGGGTGCAAGCGTTAATCGGAATTACTGGGCGTAAAGCGCACGCAGGCGGTCTGTTA
seq52           CGGAGGGTGCAAGCGTTAATCGGAATTACTGGGCGTAAAGCGCACGCAGGCGGTCTGTTA
seq19           CGGAGGGTGCAAGCGTTAATCGGAATTACTGGGCGTAAAGCGCACGCAGGCGGTCTGTTA
seq40           CGGAGGGTGCAAGCGTTAATCGGAATTACTGGGCGTAAAGCGCACGCAGGCGGTCTGTTA
seq43           CGGAGGGTGCAAGCGTTAATCGGAATTACTGGGCGTAAAGCGCACGCAGGCGGTCTGTTA
seq25           CGGAGGGTGCAAGCGTTAATCGGAATTACTGGGCGTAAAGCGCACGCAGGCGGTCTGTcA
seq1            CGGAGGGTGCAAGCGTTAATCGGAATTACTGGGCGTAAAGCGCACGCAGGCGGTCTGTcA
seq12           CGGAGGGTGCAAGCGTTAATCGGAATTACTGGGCaTAAAGCGCACGCAGGCGGTCTGTcA
seq2            CGGAGGGTGCAAGCGTTAATCGGAATTACTGGGCGTAAAGCGCACGCAGGCGGTCTGTcA
seq8            CGGAGGGTGCAAGCGTTAATCGGAATTACTGGGCGTAAAGCGCACGCAGGCGGTCTGTcA
seq56           CGGAGGGTGCAAGCGTTAATCGGAATTACTGGGCGTAAAGCGCACGCAGGCGGTCTGTcA
seq32           CGGAGGGTGCAAGCGTTAATCGGAATTACTGGGCGTAAAGCGCACGCAGGCGGTCTGTcA

seq62           AGTCgGATGTGAAATCCCCGGGCTcAACCTGGGAACTGCATTcGAAACTGGCAGGCTAGA
seq59           AGTCAGATGTGAAATCCCCGGGCTTAACCcGGGAAtTGCATcTGAtACTGGtcaGCTAGA
seq24           AGTCAGATGTGAAATCCCCGcGCTTAACgTGGGAACTGCATTTGAAACTGGCAaGCTAGA
seq27           AGTCAGATGTGAAATCCCCGcGCTTAACgTGGGAACTGCATTTGAAACTGGCAaGCTAGA
seq61           AGTCgGATGTGAAATCCCCGGGCTcAACCTGGGAACTGCATTcGAAACTGGCAGGCTAGA
seq3            AGTCAGATGTGAAATCCCCGGGCTcAACCTGGGAACTGCATTTGAAACTGGCAaGCTAGA
seq14           AGTCAGATGTGAAATCCCCGGGCTcAACCTGGGAACTGCATTTGAAACTGGCAaGCTAGA
seq20           AGTCAGATGTGAAATCCCCGGGCTTAACCTGGGAACTGCATTTGAAACTGGCAGGCTtGA
seq21           AGTCAGATGTGAAATCCCCGGGCTTAACCTGGGAACTGCATTTGAAACTGGCAGGCTtGA
seq33           AGTCAGATGTGAAATCCCCGGGCTTAACCTGGGAACTGCATTTGAAACTGGCAGGCTtGA
seq67           AGTCAGATGTGAAATCCCCGGGCTTAACCTGGGAACTGCATTcGAAACTGGCAGGCTAGA
seq35           AGTCAGATGTGAAATCCCCGGGCTTAACCTGGGAACTGCATTTGAAACTGGCAGGCTtGA
seq52           AGTCAGATGTGAAATCCCCGGGCTTAACCTGGGAACTGCATTTGAAACTGGCAGGCTtGA
seq19           AGTCAGATGTGAAATCCCCGGGCTTAACCTGGGAACTGCATTTGAAACTGGCAGGCTtGA
seq40           AGTCAGATGTGAAATCCCCGGGCTTAACCTGGGAACTGCATTTGAAACTGGCAGGCTtGA
seq43           AGTCAGATGTGAAATCCCCGGGCTTAACCTGGGAACTGCATTTGAAACTGGCAGGCTtGA
seq25           AGTCgGATGTGAAATCCCCGGGCTcAACCTGGGAACTGCATTcGAAACTGGCAGGCTAGA
seq1            AGTCgGATGTGAAATCCCCGGGCTcAACCTGGGAACTGCATTcGAAACTGGCAGGCTAGA
seq12           AGTCgGATGTGAAATCCCCGGGCTcAACCTGGGAACTGCATTcGAAACTGGCAGGCTAGA
seq2            AGTCgGATGTGAAATCCCCGGGCTcAACCTGGGAACTGCATTcGAAACTGGCAGGCTAGA
seq8            AGTCgGATGTGAAATCCCCGGGCTcAACCTGGGAACTGCATTcGAAACTGGCAGGCTAGA
seq56           AGTCgGATGTGAAATCCCCGGGCTcAACCTGGGAACTGCATTcGAAACTGGCAGGCTAGA
seq32           AGTCgGATGTGAAATCCCCGGGCTcAACCTGGGAACTGCATTcGAAACTGGCAGGCTAGA

seq62           GTCTTGTAGAGGGGGGTAGAATTCCAGGTGTAGCGGTGAAATGCGTAGAGATCTGGAGGA
seq59           GTCTTGTAGAGGGGGGTAGAATTCCAtGTGTAGCGGTGAAATGCGTAGAGATgTGGAGGA
seq24           GTCTTGTAGAGGGGGGTAGAATTCCAGGTGTAGCGGTGAAATGCGTAGAGATCTGGAGGA
seq27           GTCTTGTAGAGGGGGGTAGAATTCCAGGTGTAGCGGTGAAATGCGTAGAGATCTGGAGGA
seq61           GTCTTGTAGAGGGGGGTAGAATTCCAGGTGTAGCGGTGAAATGCGTAGAGATCTGGAGGA
seq3            GTCTcGTAGAGGGGGGTAGAATTCCAGGTGTAGCGGTGAAATGCGTAGAGATCTGGAGGA
seq14           GTCTcGTAGAGGGGGGTAGAATTCCAGGTGTAGCGGTGAAATGCGTAGAGATCTGGAGGA
seq20           GTCTcGTAGAGGGGGGTAGAATTCCAGGTGTAGCGGTGAAATGCGTAGAGATCTGGAGGA
seq21           GTCTcGTAGAGGGGGGTAGAATTCCAGGTGTAGCGGTGAAATGCGTAGAGATCTGGAGGA
seq33           GTCTTGTAGAGGGGGGTAGAATTCCAGGTGTAGCGGTGAAATGCGTAGAGATCTGGAGGA
seq67           GTCTTGTAGAGGGGGGTAGAATTCCAGGTGTAGCGGTGAAATGCGTAGAGATCTGGAGGA
seq35           GTCTTGTAGAGGGGGGTAGAATTCCAGGTGTAGCGGTGAAATGCGTAGAGATCTGGAGGA
seq52           GTCTTGTAGAGGGGGGTAGAATTCCAGGTGTAGCGGTGAAATGCGTAGAGATCTGGAGGA
seq19           GTCTcGTAGAGGGGGGTAGAATTCCAGGTGTAGCGGTGAAATGCGTAGAGATCTGGAGGA
seq40           GTCTcGTAGAGGGGGGTAGAATTCCAGGTGTAGCGGTGAAATGCGTAGAGATCTGGAGGA
seq43           GTCTcGTAGAGGGGGGTAGAATTCCAGGTGTAGCGGTGAAATGCGTAGAGATCTGGAGGA
seq25           GTCTTGTAGAGGGGGGTAGAATTCCAGGTGTAGCGGTGAAATGCGTAGAGATCTGGAGGA
seq1            GTCTTGTAGAGGGGGGTAGAATTCCAGGTGTAGCGGTGAAATGCGTAGAGATCTGGAGGA
seq12           GTCTTGTAGAGGGGGGTAGAATTCCAGGTGTAGCGGTGAAATGCGTAGAGATCTGGAGGA
seq2            GTCTTGTAGAGGGGGGTAGAATTCCAGGTGTAGCGGTGAAATGCGTAGAGATCTGGAGGA
seq8            GTCTTGTAGAGGGGGGTAGAATTCCAGGTGTAGCGGTGAAATGCGTAGAGATCTGGAGGA
seq56           GTCTTGTAGAGGGGGGTAGAATTCCAGGTGTAGCGGTGAAATGCGTAGAGATCTGGAGGA
seq32           GTCTTGTAGAGGGGGGTAGAATTCCAGGTGTAGCGGTGAAATGCGTAGAGATCTGGAGGA

seq62           ATACCGGTGGCGAAGGCGGCCCCCTGGACAAAGACTGACGCTCAGGTGCGAAAGCGTGGG
seq59           ATACCGGTGGCGAAGGCGGCCCCCTGGACAAAGACTGACGCTCAGGTGCGAAAGCGTGGG
seq24           ATACCGGTGGCGAAGGCGGCCCCCTGGACAAAGACTGACGCTCAGGTGCGAAAGCGTGGG
seq27           ATACCGGTGGCGAAGGCGGCCCCCTGGACAAAGACTGACGCTCAGGTGCGAAAGCGTGGG
seq61           ATACCGGTGGCGAAGGCGGCCCCCTGGACAAAGACTGACGCTCAGGTGCGAAAGCGTGGG
seq3            ATACCGGTGGCGAAGGCGGCCCCCTGGACgAAGACTGACGCTCAGGTGCGAAAGCGTGGG
seq14           ATACCGGTGGCGAAGGCGGCCCCCTGGACgAAGACTGACGCTCAGGTGCGAAAGCGTGGG
seq20           ATACCGGTGGCGAAGGCGGCCCCCTGGACgAAGACTGACGCTCAGGTGCGAAAGCGTGGG
seq21           ATACCGGTGGCGAAGGCGGCCCCCTGGACgAAGACTGACGCTCAGGTGCGAAAGCGTGGG
seq33           ATACCGGTGGCGAAGGCGGCCCCCTGGACAAAGACTGACGCTCAGGTGCGAAAGCGTGGG
seq67           ATACCGGTGGCGAAGGCGGCCCCCTGGACAAAGACTGACGCTCAGGTGCGAAAGCGTGGG
seq35           ATACCGGTGGCGAAGGCGGCCCCCTGGACAAAGACTGACGCTCAGGTGCGAAAGCGTGGG
seq52           ATACCGGTGGCGAAGGCGGCCCCCTGGACAAAGACTGACGCTCAGGTGCGAAAGCGTGGG
seq19           ATACCGGTGGCGAAGGCGGCCCCCTGGACgAAGACTGACGCTCAGGTGCGAAAGCGTGGG
seq40           ATACCGGTGGCGAAGGCGGCCCCCTGGACgAAGACTGACGCTCAGGTGCGAAAGCGTGGG
seq43           ATACCGGTGGCGAAGGCGGCCCCCTGGACgAAGACTGACGCTCAGGTGCGAAAGCGTGGG
seq25           ATACCGGTGGCGAAGGCGGCCCCCTGGACAAAGACTGACGCTCAGGTGCGAAAGCGTGGG
seq1            ATACCGGTGGCGAAGGCGGCCCCCTGGACAAAGACTGACGCTCAGGTGCGAAAGCGTGGG
seq12           ATACCGGTGGCGAAGGCGGCCCCCTGGACAAAGACTGACGCTCAGGTGCGAAAGCGTGGG
seq2            ATACCGGTGGCGAAGGCGGCCCCCTGGACAAAGACTGACGCTCAGGTGCGAAAGCGTGGG
seq8            ATACCGGTGGCGAAGGCGGCCCCCTGGACAAAGACTGACGCTCAGGTGCGAAAGCGTGGG
seq56           ATACCGGTGGCGAAGGCGGCCCCCTGGACAAAGACTGACGCTCAGGTGCGAAAGCGTGGG
seq32           ATACCGGTGGCGAAGGCGGCCCCCTGGACAAAGACTGACGCTCAGGTGCGAAAGCGTGGG

seq62           GAGCAAA
seq59           GAGCAAA
seq24           GAGCAAA
seq27           GAGCAAA
seq61           GAGCAAA
seq3            GAGCAAA
seq14           GAGCAAA
seq20           GAGCAAA
seq21           GAGCAAA
seq33           GAGCAAA
seq67           GAGCAAA
seq35           GAGCAAA
seq52           GAGCAAA
seq19           GAGCAAA
seq40           GAGCAAA
seq43           GAGCAAA
seq25           GAGCAAA
seq1            GAGCAAA
seq12           GAGCAAA
seq2            GAGCAAA
seq8            GAGCAAA
seq56           GAGCAAA
seq32           GAGCAAA
```
